# TreeVector: Scalable, Interactive, Phylogenetic Trees for the Web

**DOI:** 10.1371/journal.pone.0008934

**Published:** 2010-01-28

**Authors:** Ralph Pethica, Gary Barker, Tim Kovacs, Julian Gough

**Affiliations:** 1 Computer Science Department, University of Bristol, Bristol, United Kingdom; 2 School of Biological Sciences, University of Bristol, Bristol, United Kingdom; Georgia Institute of Technology, United States of America

## Abstract

**Background:**

Phylogenetic trees are complex data forms that need to be graphically displayed to be human-readable. Traditional techniques of plotting phylogenetic trees focus on rendering a single static image, but increases in the production of biological data and large-scale analyses demand scalable, browsable, and interactive trees.

**Methodology/Principal Findings:**

We introduce TreeVector, a Scalable Vector Graphics–and Java-based method that allows trees to be integrated and viewed seamlessly in standard web browsers with no extra software required, and can be modified and linked using standard web technologies. There are now many bioinformatics servers and databases with a range of dynamic processes and updates to cope with the increasing volume of data. TreeVector is designed as a framework to integrate with these processes and produce user-customized phylogenies automatically. We also address the strengths of phylogenetic trees as part of a linked-in browsing process rather than an end graphic for print.

**Conclusions/Significance:**

TreeVector is fast and easy to use and is available to download precompiled, but is also open source. It can also be run from the web server listed below or the user's own web server. It has already been deployed on two recognized and widely used database Web sites.

## Introduction

Producing a graphical visualisation of a phylogenetic tree is a common need of researchers involved in studies of computational biology and molecular evolution. Phylogenies can be inferred from many types of data, e.g. protein/DNA alignments, results of expression clustering and Interpro hierarchies, using specialist phylogenetics packages such as Phylip [1], Paup [2] and others. The phylogenies produced by these software packages and algorithms are normally saved in a computer readable format for further computational analysis, or human inspection using tree visualisation software. One of the simplest and most common data formats for representation of a tree topology is the Newick [3] parenthesis standard. This format is an effective way of storing information on branch lengths, node names and topology in a compressed and easy to parse format. Once a tree topology is produced, if visual analysis is required, it may be plotted in various ways depending on the requirements of the user. Some users simply require a graphic for use in a publication or a slide, and for others there is often a demand to investigate one large tree in great detail or alternatively to look briefly at many different trees.

We introduce TreeVector, a Java based phylogenetic visualisation tool which aims to address the diverse and changing needs of the user and take advantage of the standards and technologies which are appropriate to this type of graphical visualisation.

## Results

**Figure 1:**
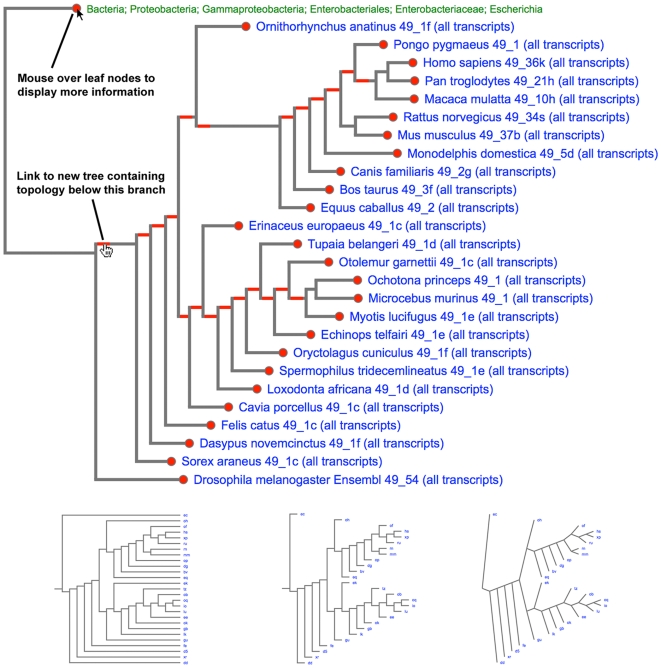
A view of the TreeVector web interface integrated with the SUPERFAMILY server. Interactive features of TreeVector are shown in Figure 1. Moving the mouse over the red circles displays extended information, such as taxonomy instead of organism name. Red links in branches can be used to display a tree containing only the nodes below this branch. The blue leaf labels link to the relevant genome pages on SUPERFAMILY. Also shown are some of the other formats of tree output available, such as triangular branches and aligned labels.

TreeVector uses Scalable Vector Graphics (SVG) as the primary output. SVG is an XML format for showing two-dimensional vector graphics, and is supported natively by the majority of modern web browsers. This format is well suited to online visualisation in phylogenetics, as it can be manipulated and explored in a web browser, produces small files (for a tree of 677 genomes, file size is 324k for SVG compared to 1.6 megabytes for a PNG), and can be animated and linked using standard web scripts such as Javascript and CSS (See Figure 1).

Broadly speaking TreeVector has the following advantages over existing systems:

- It can be used locally on the command line or implemented as server software and accessed through a web page.
- Information in trees can be linked to other pages via standard hyperlinks.
- Visualisation is displayed using SVG graphical format allowing for smaller filesize and download time, as well as user customisation through CSS stylesheets and Javascript.
- SVG is a vector format, and allows trees to be zoomed and panned in a web browser with no loss of quality.
- Database access and Javascript allows further, dynamic information to be embedded into the trees.
- Very large trees can be created without producing huge files.
- SVG is supported by graphic editors such as Inkscape, making annotation and application of visual effects to downloaded trees easy.

There are many different visualisation tools for phylogenetics, and it is not uncommon for individual research groups to write their own plotter, specific to their requirements. With this in mind TreeVector has been designed as a flexible framework, which can be quickly integrated into servers or an automated process for the production of user tailored phylogenies. The majority of existing tree plotting software is designed for local installation and viewing, for example packages such as TREEVIEW [4], ATV [5], MacClade [6]. Many of the phylogenetic inference packages have built in visualisation methods, such as Phylip's Drawtree module, and Paup's text based tree output.

These local installations have the advantage that an internet connection is not needed, and there is potential for a high degree of customisation of the final graphic. The trees can also be manipulated in various ways in order to highlight specific trends. However, such packages are often platform dependent, and not suitable for a user who wants to quickly view a tree and move on to other data. New trees must be loaded manually into the program, and it is difficult to link from specific parts of the tree to other related content.

There are several tree plotting programs that have a web interface, allowing the user to directly input tree topologies and display the output in a web page. Phylodendron [7] and PHY.FI [8] are two such implementations, other web versions include T-REX [9], iTOL[10] and Drawtree [11]. These have the advantage that no software needs to be installed and they can be used by any computer connected to the internet. Some of these are able to produce an SVG output, though it is given as a download format rather than an embedded graphic, with the exception of PhyloView [12], which uses SVG natively. PhyloView however is designed as a protein sequence and taxonomy comparison tool rather than a phylogenetic tree generator.

An extra feature of TreeVector is a script which allows specific names to be extracted from the topology and displayed on their own, using the relationships taken from the parent tree topology. This can be very useful when a user is only interested in specific parts of the tree, and avoids the need of a separate phylogenetic inference from the data. This approach can also help provide a greater level of accuracy for smaller selections as relationships are based on larger datasets.

## Methods

TreeVector is written in Java and can be integrated into a server using a series of Perl scripts. Scalable Vector Graphics (SVG) is the native output format for all graphics produced by TreeVector, though Inkscape [13] is used to convert vector graphics to other formats, including PDF, JPG and PNG. The output tree can also be saved as XML format. Preference files can be set up to control most features, such as output location and database access. The SVG output trees reference a Javascript file which can be modified to add dynamic features, and a CSS file to control the visual details.

TreeVector is integrated into the SUPERFAMILY [14] server, a database of structural protein domain annotations for all completely sequenced organisms. A phylogenetic tree based on domain architectures is produced and regularly updated on the SUPERFAMILY server using PAUP. TreeVector is used to dynamically generate images for visualising and browsing the stored tree (e.g. http://supfam.cs.bris.ac.uk/SUPERFAMILY/cgi-bin/genome_names.cgi). Since SVGs are scalable, the whole tree containing hundreds of organisms can be examined by zooming and panning. Alternatively any subset of genomes can be selected and their phylogeny is parsed out of the stored tree and an image dynamically generated by TreeVector including only those of interest. In this way only the display is restricted to nodes of interest, but the accuracy undiminished since the data from all organisms is used every time; the tree topology is not recalculated using only the reduced subset of organisms. SVGs can be interactive, and further information on NCBI taxonomy is available by rolling over leaf nodes, and leaf labels are links to the relevant genome pages on the SUPERFAMILY server. The cached topology for the full tree allows for very rapid browsing as computationally expensive phylogenetic inference does not need to be carried out for each selection. A feature has also been added which includes a hyperlink in each node of the tree which, when clicked will display a tree containing just the genomes below that node (See Figure 1).

The functional genomics group at Bristol University host a database of wheat SNP data, produced by autoSNP [15]. TreeVector is used alongside Quicktree [16] to produce trees showing the phylogenetic relationships of different wheat varieties and homoeologous gene sequences. In this process thousands of trees are produced and stored each time the server is updated. Storing the trees as SVG means that far less disk space is used for the graphics compared to bitmapped alternatives, the download size is smaller, cutting down on server bandwidth requirements, and rendering is done at the client side, decreasing the processing power needed on the server (e.g. http://bio-sanger.bio.bris.ac.uk/SNP/wheat/snp_1.htm).

## Discussion

### Conclusions

TreeVector is a robust, open source software product for the biological community. Phylogenetic trees can be plotted from data files generated from popular software using the NEXUS format producing scalable vector graphics. TreeVector represents a significant advance on existing software, making use of standards and technologies which may not have been established when previous products were developed. Specifically TreeVector offers new levels of flexibility and interactiveness lending itself well to dynamic web-based implementations.

TreeVector has already been deployed in two recognised databases, dynamically displaying domain-based organism trees and trees for wheat SNP data. It is expected that this software will be of widespread use to the bioinformatics community.

### Availability and Requirements

Project name: TreeVector

Project home page: http://supfam.cs.bris.ac.uk/TreeVector/

Operating system(s): platform independent

Programming language: Java

Other requirements: an SVG-enabled browser

License: GNU GPL

Any restrictions to non-academics: none
